# Influence of Health Beliefs on Adherence to COVID-19 Preventative Practices: International, Social Media–Based Survey Study

**DOI:** 10.2196/23720

**Published:** 2021-02-26

**Authors:** Julianna C Hsing, Jasmin Ma, Alejandra Barrero-Castillero, Shilpa G Jani, Uma Palam Pulendran, Bea-Jane Lin, Monika Thomas-Uribe, C Jason Wang

**Affiliations:** 1 Department of Epidemiology and Population Health Stanford University School of Medicine Stanford, CA United States; 2 Center for Policy, Outcomes, and Prevention Department of Pediatrics Stanford University School of Medicine Stanford, CA United States; 3 Division of Neonatology Beth Israel Deaconess Medical Center Boston, MA United States; 4 Division of Newborn Medicine Boston Children's Hospital Boston, MA United States; 5 Department of Pediatrics Harvard Medical School Boston, MA United States; 6 Department of Pediatrics University of California San Francisco - Fresno Fresno, CA United States; 7 Center for Health Policy Freeman-Spogli Institute for International Studies Stanford University Stanford, CA United States; 8 Center for Primary Care Outcomes Research Stanford University School of Medicine Stanford, CA United States

**Keywords:** COVID-19 pandemic, health belief model, behavior change, preventative health behaviors, handwashing, social distancing, international, online survey, social media, cross-sectional study

## Abstract

**Background:**

Health behavior is influenced by culture and social context. However, there are limited data evaluating the scope of these influences on COVID-19 response.

**Objective:**

This study aimed to compare handwashing and social distancing practices in different countries and evaluate practice predictors using the health belief model (HBM).

**Methods:**

From April 11 to May 1, 2020, we conducted an online, cross-sectional survey disseminated internationally via social media. Participants were adults aged 18 years or older from four different countries: the United States, Mexico, Hong Kong (China), and Taiwan. Primary outcomes were self-reported handwashing and social distancing practices during COVID-19. Predictors included constructs of the HBM: perceived susceptibility, perceived severity, perceived benefits, perceived barriers, self-efficacy, and cues to action. Associations of these constructs with behavioral outcomes were assessed by multivariable logistic regression.

**Results:**

We analyzed a total of 71,851 participants, with 3070 from the United States, 3946 from Mexico, 1201 from Hong Kong (China), and 63,634 from Taiwan. Of these countries, respondents from the United States adhered to the most social distancing practices (χ^2^_3_=2169.7, *P*<.001), while respondents from Taiwan performed the most handwashing (χ^2^_3_=309.8, *P*<.001). Multivariable logistic regression analyses indicated that self-efficacy was a positive predictor for handwashing (odds ratio [OR]_United States_ 1.58, 95% CI 1.21-2.07; OR_Mexico_ 1.5, 95% CI 1.21-1.96; OR_Hong Kong_ 2.48, 95% CI 1.80-3.44; OR_Taiwan_ 2.30, 95% CI 2.21-2.39) and social distancing practices (OR_United States_ 1.77, 95% CI 1.24-2.49; OR_Mexico_ 1.77, 95% CI 1.40-2.25; OR_Hong Kong_ 3.25, 95% CI 2.32-4.62; OR_Taiwan_ 2.58, 95% CI 2.47-2.68) in all countries. Handwashing was positively associated with perceived susceptibility in Mexico, Hong Kong, and Taiwan, while social distancing was positively associated with perceived severity in the United States, Mexico, and Taiwan.

**Conclusions:**

Social media recruitment strategies can be used to reach a large audience during a pandemic. Self-efficacy was the strongest predictor for handwashing and social distancing. Policies that address relevant health beliefs can facilitate adoption of necessary actions for preventing COVID-19. Our findings may be explained by the timing of government policies, the number of cases reported in each country, individual beliefs, and cultural context.

## Introduction

The severity and rapid transmission of COVID-19 has forced most regions to implement community mitigation strategies. These strategies have ranged from government guidelines on personal protective measures and social distancing to strict lockdown orders that closed schools and businesses [1]. Nationwide school closures in 194 countries in early April 2020 demonstrated the extent of these interventions [2]. These measures have reduced transmission or delayed the peak of infection of past pandemics to varying degrees, which were estimated to have prevented at least 60 million COVID-19 cases [3,4].

Although these interventions reduce the stress on health care systems, they also incur high economic and societal costs, making adherence more difficult for those under financial strain [3,5]. Recent studies have begun to assess adherence to COVID-19 guidelines, evaluating demographic characteristics and the impact of guideline duration [6-8]. Some have suggested that concepts from social and behavioral sciences can provide insight into adherence to guidelines, but current data evaluating these hypotheses in multiple countries and in the context of COVID-19 using relevant behavior change theories, such as the health belief model (HBM), are limited [9]. Given the rapid spread of COVID-19 and the scale of guidelines worldwide, a cross-cultural assessment of preventative health behaviors is essential to identifying which approaches improve adherence. This study aims to compare handwashing and social distancing behaviors across four different countries using the HBM.

## Methods

### Participant Recruitment

From April 11 to May 1, 2020, we conducted a confidential, cross-sectional, international open survey through the following social media platforms: Facebook, Instagram, Line, and Twitter. The survey was announced and advertised through Stanford Health Policy’s social media accounts. Facebook boosted posts were used to target social media users who were 18 years of age or older. We focused our analysis on countries and regions with at least 1000 survey responses: the United States, Mexico, Hong Kong (China), and Taiwan. Facebook is the most popular social media platform among adults in all four countries, whereas Instagram, Twitter, and Line have relatively high penetration in specific groups and countries [10-13]. Though the limitations of using convenience sampling and social media are well-known, this method is cost-effective, time-efficient, and most feasible for reaching a large international audience in a fast-spreading pandemic [14]. The alternative of administering telephone surveys is associated with extremely low response rates (6% in 2018) and limitations on item complexity and survey length [15,16].

The survey was developed on Qualtrics (Qualtrics Inc), an online survey distribution tool, and administered in English, Spanish, and Mandarin. Translations were provided by native speakers fluent in the respective languages, who tested the survey before it was fielded. Prior to survey completion, participants were provided with information about the study and were asked to acknowledge consent to the study. All items were optional except for country of residence. Through Qualtrics, cookies were used to assign a unique user identifier to each client computer to prevent participants from completing the survey more than once. Only completed surveys were analyzed. Given that no incentives were offered to participants and that the survey was voluntary, we did not assess whether surveys were completed in an atypical amount of time. The study was reviewed and approved by Stanford University’s Institutional Review Board.

### Conceptual Model and Survey Items

**Figure 1:**
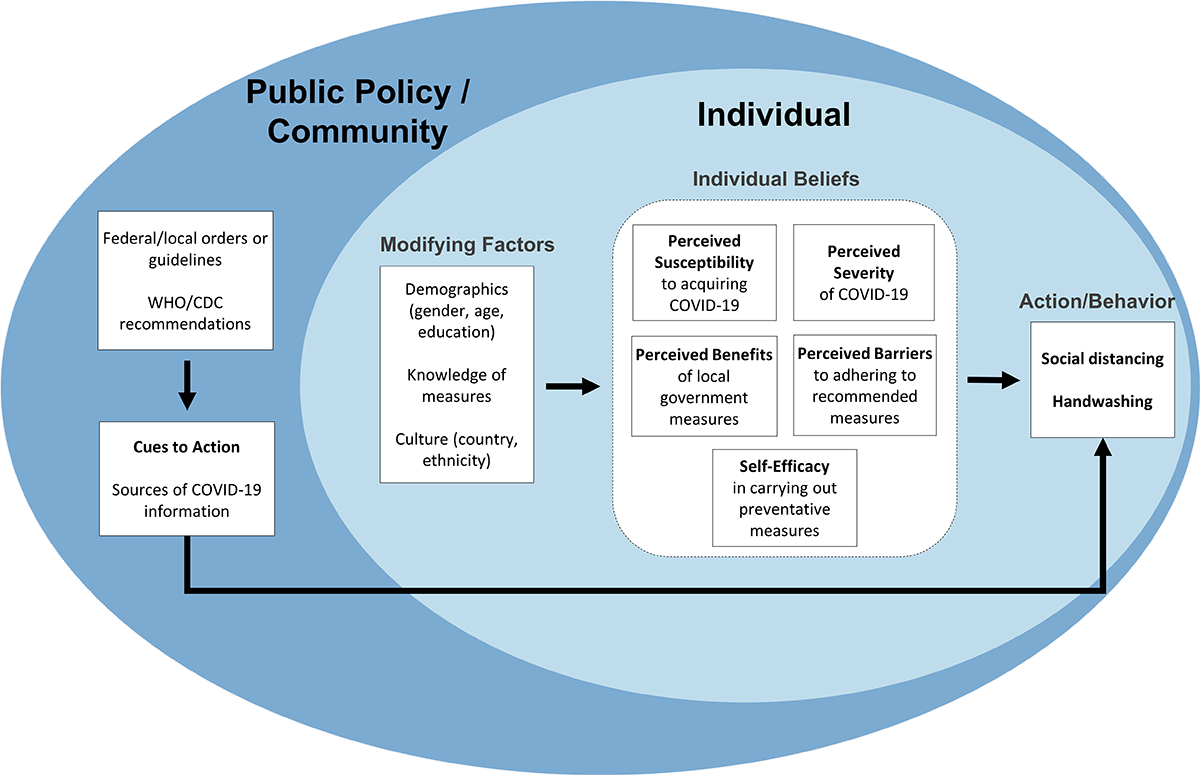
Conceptual framework of the study adapted from the health belief model to assess individual health beliefs, modifying factors, and the effects of public policy on social distancing and handwashing behaviors during the COVID-19 pandemic. CDC: Centers for Disease Control and Prevention; WHO: World Health Organization.

We used the HBM, a widely used framework for explaining health behaviors and guiding related interventions, to create survey items to assess health beliefs among respondents in the four countries [17]. The HBM accounts for both individual- and community-level factors of health motivation, making it an ideal option for addressing health behavior problems that evoke health concerns during the COVID-19 pandemic. Figure 1 shows the key constructs in the HBM that determine behavior, including individual beliefs (ie, perceived susceptibility, severity, benefits, and barriers as well as self-efficacy), which may be influenced by sociodemographic factors or knowledge, and cues to action, which may be influenced by public policy.

Survey items (see Multimedia Appendix 1) were developed based on prior expertise, survey knowledge, and group discussion. We asked participants the following questions for each HBM construct:

1. Perceived susceptibility. What do you think your risk is of getting infected with COVID-19?
2. Perceived severity. How afraid are you of the COVID-19 pandemic?
3. Perceived benefits. How do you feel about the government measures of COVID-19 in your area?
4. Perceived barriers. Have any barriers prevented you from adhering to measures in your area?
5. Self-efficacy. How confident are you that you are able to and willing to carry out these measures?
6. Cues to action. What are your sources of information regarding COVID-19?

Perceived susceptibility, perceived severity, and self-efficacy items were assessed using a 5-point Likert scale. During analysis, response scales were eventually collapsed into three categories, such as *not likely/slightly likely*, *moderately likely*, and *likely/very likely*. Perceived benefit items were assessed using three categories: *unnecessarily restrictive/moderately restrictive*, *essential/appropriate*, and *not enough*. Perceived barriers and cues to action were both assessed on a binary *yes/no* scale.

To account for modifying factors that influenced individual beliefs, we assessed for age (ie, 18-24, 25-34, 35-44, 45-59, and 60+ years), gender (ie, male, female, and other), highest educational attainment (ie, high school or less and college and above), country of residence (ie, United States, Mexico, Hong Kong, and Taiwan), race or ethnicity (eg, Asian, Hispanic/Latino, and White or European), change in income due to COVID-19 (ie, yes or no), and awareness of government measures or guidelines (ie, some/not aware and most/all).

Handwashing behaviors were assessed by asking respondents whether they washed their hands or used hand sanitizer in the following seven situations: (1) after coming home from being outside; (2) after grocery shopping; (3) after interacting with nonhousehold members; (4) while being in public; (5) before or after using their vehicle; (6) after blowing their nose, coughing, or sneezing into their hand; and (7) before eating. Responses for all situations were summed up to a score of 7. Social distancing behaviors were evaluated by assessing whether respondents did the following: (1) avoided nonessential gatherings, (2) kept at least the recommended distance from nonhousehold members (eg, 6 feet, 1.5 meters, 2 meters, etc), or (3) avoided close contact with individuals at higher risk for severe illness from COVID-19. Responses were summed up to a score of 3. Total adherence to either handwashing or social distancing responses was assessed by a binary variable, with individuals performing all of the practices as one group (yes = 1) and those who performed fewer than all practices as the other group (no = 0); this was done for each of the two behaviors.

### Statistical Analysis

We conducted poststratification weighting for each country by age and gender—and race or ethnicity for the United States—using each country’s most recent census data [18-21]. Weights were calculated by dividing each stratified proportion of the country’s population by each stratified proportion of the study’s country sample, followed by renormalizing for each country to ensure that weighted sample size equaled the unweighted sample size [22]. Weighted frequencies and percentages were calculated for categorical variables and compared using chi-square tests. To assess which country performed more handwashing and social distancing practices, countries were analyzed together in multivariate analyses, with each country coded as a key independent dichotomous variable, adjusting for gender, age, education, and reduced income.

Countries were also analyzed separately with multivariable logistic regressions to examine the association of HBM constructs with two main outcomes: handwashing and social distancing practices. HBM covariates included perceived susceptibility, severity, benefits, and barriers; self-efficacy; and cues to action. All models were adjusted for gender, age, education, and reduced income. To ensure our handwashing variable appropriately captured COVID-19-related handwashing behaviors, we also ran a sensitivity analysis that assessed the association between handwashing time (ie, >20 seconds vs ≤20 seconds) and HBM constructs, because this handwashing duration was a specific COVID-19 recommendation in all four countries [23-26]. For all models, odds ratios (ORs) and 95% CIs were calculated. All statistical analyses were performed using R statistical software, version 3.6.3 (The R Foundation), and *P* values were 2-sided with an α of .05.

## Results

### Participant Characteristics

A total of 71,851 individuals were included in our analysis: 3070 from the United States (4.3%), 3946 from Mexico (5.5%), 1201 from Hong Kong (1.7%), and 63,634 from Taiwan (88.6%). Of these, 71,728 (99.8%) completed at least 80% of the survey (14 of 17 questions). Missing data for each item were less than 5% and, thus, were not imputed. After weighting, the gender and age distributions were representative of each country according to their most recent census data (see Multimedia Appendices 2 and 3). Overall, Mexico had a younger population compared to other countries, and most respondents in all four countries had a college degree or higher. A total of 2099 out of 3931 respondents (53.4%) from Mexico, 337 out of 1189 (28.3%) from Hong Kong, 779 out of 3062 (25.4%) from the United States, and 10,725 out of 63,399 (16.9%) from Taiwan reported reduced income due to COVID-19. A total of 68,614 out of 71,633 respondents (95.8%) in all countries were aware of government measures and/or guidelines (see Table 1).

**Table 1:** Weighted demographic characteristics of survey respondents by country.

Characteristic			Value (N=71,851), n (%)^a^								*P* value^b^
			United States (n=3070)		Mexico (n=3946)		Hong Kong (n=1201)		Taiwan (n=63,634)		
**Age group (years)**											<.001
	18-24	110 (3.6)		507 (12.9)		83 (7.0)		4969 (7.8)		—^c^	
	25-34	519 (17.0)		953 (24.2)		198 (16.6)		10,509 (16.6)		—	
	35-44	451 (14.7)		820 (20.8)		226 (18.9)		12,865 (20.3)		—	
	45-59	963 (31.4)		977 (24.8)		310 (25.9)		17,836 (28.1)		—	
	60+	1019 (33.3)		684 (17.3)		377 (31.6)		17,294 (27.2)		—	
**Gender**											<.001
	Female	1683 (55.0)		2031 (51.6)		602 (50.4)		31,407 (49.6)		—	
	Male	1351 (44.2)		1867 (47.4)		562 (47.1)		30,034 (47.4)		—	
	Other^d^	25 (0.8)		40 (1.0)		30 (2.5)		1894 (3.0)		—	
**Race or ethnicity**											<.001
	Asian	158 (5.2)		15 (0.4)		1180 (98.9)		62,924 (99.3)		—	
	Hispanic/Latino or other^e^	1520 (49.7)		3319 (84.7)		11 (0.9)		228 (0.4)		—	
	White or European	1379 (45.1)		586 (15.0)		2 (0.2)		207 (0.3)		—	
**Education**											<.001
	Below college	286 (9.4)		726 (18.4)		263 (22.2)		7889 (12.4)		—	
	College and above	2768 (90.6)		3215 (81.6)		923 (77.8)		55,547 (87.6)		—	
**Reduced income since COVID-19**											<.001
	No	2283 (74.6)		1832 (46.6)		852 (71.7)		52,674 (83.1)		—	
	Yes	779 (25.4)		2099 (53.4)		337 (28.3)		10,725 (16.9)		—	
**Awareness of governmental measures and/or guidelines**											<.001
	Some/not aware	20 (0.7)		123 (3.1)		26 (2.2)		2850 (4.5)		—	
	All/most	3034 (99.3)		3818 (96.9)		1167 (97.8)		60,595 (95.5)		—	

^a^Weighted values were calculated by dividing the actual proportion of the country’s population by the proportion from the study’s sample, then renormalized for each country to ensure weighted and unweighted sample sizes were equal. Due to rounding and missing data (<5% for each item), the sum of frequencies and percentages for the sample weighted columns may not equal the country’s total sample size.

^b^*P* values were calculated using 2-sided chi-square tests.

^c^Not available.

^d^Responses of *other* gender include individuals who chose nonbinary/third gender, prefer not to say, or other (<3% of total responses).

^e^Responses of *other* race or ethnicity include individuals who are African, Black, African American, American Indian or Alaskan Native, Middle Eastern, Native Hawaiian or other Pacific Islander, or other. Categories were collapsed due to low numbers (<2% of total responses).

### Handwashing and Social Distancing Behaviors

**Figure 2:**
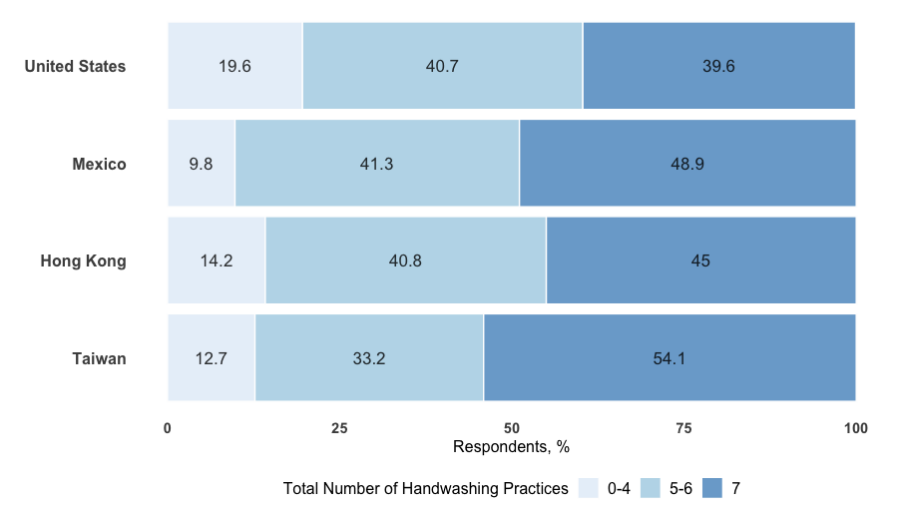
Distribution of handwashing practices by country. Respondents were asked whether they washed their hands or used hand sanitizer in any of the following seven situations: (1) after coming home from being outside; (2) after grocery shopping; (3) after interacting with nonhousehold members; (4) while being in public; (5) before or after using their vehicle; (6) after blowing their nose, coughing, or sneezing into their hand; and (7) before eating.

**Figure 3:**
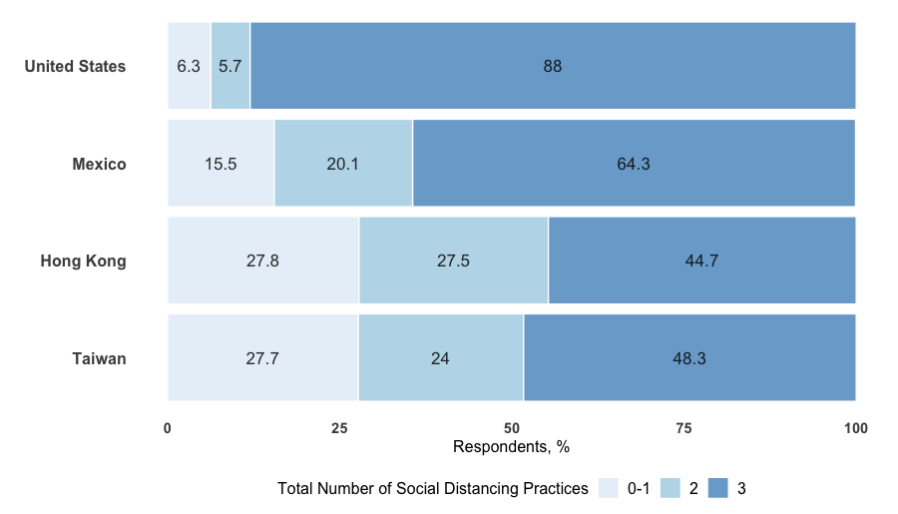
Distribution of social distancing practices by country. Respondents were asked whether they did the following: (1) avoided nonessential gatherings, (2) kept at least the recommended healthy distance from nonhousehold members (eg, 6 feet, 1.5 meters, and 2 meters), or (3) avoided close contact with individuals at higher risk for severe illness from COVID-19.

Bivariate chi-square analyses showed that respondents from Taiwan practiced the most handwashing behaviors (χ^2^_3_=309.8, *P*<.001) relative to other countries, while those from the United States practiced the most social distancing (χ^2^_3_=2169.7, *P*<.001). Of the 71,608 respondents who provided a response to their handwashing practices, 39.6% (1215/3066) from the United States, 48.8% (1927/3938) from Mexico, 45% (538/1195) from Hong Kong, and 54.1% (34,328/63,409) from Taiwan reported handwashing in all seven situations (see Figure 2). Of the 71,851 respondents who provided a response to their social distancing practices, 88.0% (2702/3070) from the United States, 64.3% (2539/3946) from Mexico, 44.7% (537/1201) from Hong Kong, and 48.3% (30,737/63,634) from Taiwan reported performing all three social distancing practices (see Figure 3). We found similar patterns of association in the sensitivity multivariate analysis (see Multimedia Appendix 4). Respondents from the United States (OR 0.50, 95% CI 0.46-0.54), Mexico (OR 0.87, 95% CI 0.81-0.93), and Hong Kong (OR 0.67, 95% CI 0.59-0.75) were less likely to perform handwashing compared to those from Taiwan (reference value). Respondents from the United States (OR 7.73, 95% CI 6.93-8.66) and Mexico (OR 2.17, 95% CI 2.02-2.33) were more likely to practice social distancing compared to those from Taiwan. In contrast, respondents from Hong Kong were less likely to practice social distancing compared to those from Taiwan, although the association was only slightly significant (OR 0.88, 95% CI 0.87-0.99).

### Health Belief Model Constructs

Table 2 compares the distribution of responses to health belief questions by country, which were assessed using chi-square tests and were all statistically different across each country (*P*<.001). For perceived susceptibility, the percentage of respondents who felt they were likely (ie, moderately likely to very likely) to be infected with COVID-19 was higher in the United States (1683/3068, 54.9%) and Mexico (2688/3941, 68.2%) compared to those from Hong Kong (557/1192, 46.7%) and Taiwan (19,080/63,425, 30.1%). For perceived severity, a higher percentage of individuals from Taiwan (28,082/63,470, 44.2%) were not afraid (ie, slightly afraid or not afraid) of the COVID-19 pandemic compared to those from the United States (876/3064, 28.6%), Mexico (1154/3940, 29.3%), and Hong Kong (289/1195, 24.2%). For perceived benefits, most individuals in the United States (2062/3016, 68.4%) and Taiwan (53,573/62,625, 85.5%) believed that the government measures in place were appropriate or essential, while most individuals in Mexico (2256/3871, 58.3%) and Hong Kong (745/1172, 63.6%) believed that their measures were not enough. For self-efficacy, a majority of individuals in all countries were confident or very confident in their ability to carry out preventative measures: 88.5% (2712/3066) in the United States, 91.4% (3599/3940) in Mexico, 79.3% (946/1193) in Hong Kong, and 75.8% (48,144/63,481) in Taiwan.

Regarding perceived barriers to social distancing, Mexico (2547/3946, 64.5%) had the highest proportion of individuals who perceived difficulty in obtaining face masks, followed by 60.5% (1856/3070) of individuals in the United States, 52.7% (633/1201) in Hong Kong, and 12.2% (7736/63,634) in Taiwan. Having an essential job (eg, grocery store worker), as perceived by the individual or determined by local governments, was a common perceived barrier in all countries. Other common barriers included family obligations in Mexico as well as transportation needs in Hong Kong and Taiwan. For handwashing barriers, respondents from the United States (1536/3070, 50.0%) and Mexico (2056/3946, 52.1%) perceived more difficulty in obtaining hand sanitizer compared to those in Taiwan (2708/63,634, 4.3%) and Hong Kong (104/1201, 8.7%). Only a small proportion of individuals in all countries (<5% each) reported having difficulty obtaining hand soap.

For cues to action, respondents selected up to three sources of information for COVID-19. News (eg, TV news, newspaper, and radio) and social media were the most frequently reported sources of information in every country, with Hong Kong reporting the highest percentage (news: 1002/1201, 83.4%; social media: 846/1201, 70.4%) and Mexico the lowest (news: 1965/3946, 49.8%; social media: 1337/3946, 33.9%). More respondents selected federal, or central, government rather than regional government as a top information source in Taiwan (14,730/63,634, 23.1% vs 1387/63,634, 2.2%) and Mexico (1155/3946, 29.3% vs 567/3946, 14.4%), while more respondents selected regional rather than federal government in the United States (1129/3070, 36.8% vs 312/3070, 10.2%). Both choices were comparably low in Hong Kong (<5% each).

**Table 2:** Weighted responses to health belief model (HBM) constructs by country.

HBM construct, survey question, and responses		Value (N=71,851), n (%)^a^				*P* value^b^	
		United States (n=3070)	Mexico (n=3946)	Hong Kong (n=1201)	Taiwan (n=63,634)		
**Perceived susceptibility to infection: What do you think is your risk of getting infected with COVID-19?**							<.001
	Not likely/slightly likely	1385 (45.1)	1253 (31.8)	635 (53.3)	44,345 (69.9)		—^c^
	Moderately likely	1024 (33.4)	1523 (38.6)	331 (27.7)	10,038 (15.8)		—
	Likely/very likely	659 (21.5)	1165 (29.6)	226 (19.0)	9042 (14.3)		—
**Perceived severity of COVID-19: How afraid are you of the COVID-19 pandemic?**							<.001
	Not afraid/slightly afraid	876 (28.6)	1154 (29.3)	289 (24.2)	28,082 (44.2)		—
	Moderately afraid	1022 (33.3)	1135 (28.8)	374 (31.3)	15,216 (24.0)		—
	Afraid/very afraid	1166 (38.1)	1651 (41.9)	532 (44.5)	20,172 (31.8)		—
**Perceived benefits of measures: How do you feel about the government measures for COVID-19 in your area?**							<.001
	Appropriate/essential	2062 (68.4)	1343 (34.7)	288 (24.5)	53,573 (85.5)		—
	Unnecessarily restrictive/moderately restrictive	297 (9.9)	272 (7.0)	139 (11.9)	3227 (5.2)		—
	Not enough	657 (21.8)	2256 (58.3)	745 (63.6)	5825 (9.3)		—
**Self-efficacy in carrying out measures: How confident are you that you are able and willing to carry out these measures?**							<.001
	Not confident/slightly confident	138 (4.5)	93 (2.3)	52 (4.4)	3867 (6.1)		—
	Moderately confident	216 (7.0)	248 (6.3)	195 (16.3)	11,470 (18.1)		—
	Confident/very confident	2712 (88.5)	3599 (91.4)	946 (79.3)	48,144 (75.8)		—
**Perceived barriers to carrying out measures: Have any barriers prevented you from adhering to these measures? Do you have any difficulty getting masks, hand soap, and hand sanitizer?**							
	Masks	1856 (60.5)	2547 (64.5)	633 (52.7)	7736 (12.2)		<.001
	Essential job	381 (12.4)	630 (16.0)	333 (27.8)	16,141 (25.4)		<.001
	Family obligations	201 (6.6)	636 (16.1)	92 (7.7)	5188 (8.2)		<.001
	Transportation needs	49 (1.6)	193 (4.9)	595 (49.6)	15,158 (23.8)		<.001
	Hand soap	142 (4.6)	167 (4.2)	13 (1.1)	384 (0.6)		<.001
	Hand sanitizer	1536 (50.0)	2056 (52.1)	104 (8.7)	2708 (4.3)		<.001
**Cues to action: What are your top three sources of information regarding COVID-19?^d^**							
	News source	2119 (69.0)	1965 (49.8)	1002 (83.4)	50,443 (79.3)		<.001
	Social media	1234 (40.2)	1337 (33.9)	846 (70.4)	39,251 (61.7)		<.001
	Central administration officials	312 (10.2)	1155 (29.3)	25 (2.1)	14,730 (23.1)		<.001
	Regional administration officials	1129 (36.8)	567 (14.4)	60 (5.0)	1387 (2.2)		<.001

^a^Weighted values were calculated by dividing the actual proportion of the country’s population by the proportion from the study’s sample, then renormalized for each country to ensure weighted and unweighted sample sizes were equal. Due to rounding and missing data (<5% for each item), the sum of frequencies and percentages for the sample weighted columns may not equal the country’s total sample size.

^b^*P* values were calculated using 2-sided chi-square tests.

^c^Not available.

^d^The top four media resources selected by respondents, when asked to pick their top three from the list, are shown.

### Association of HBM Constructs With Handwashing and Social Distancing Behaviors

In multivariable analyses, individuals with higher self-efficacy were more likely to perform more handwashing practices compared to those with lower self-efficacy (OR_United States_ 1.58, 95% CI 1.21-2.07; OR_Mexico_ 1.54, 95% CI 1.21-1.96; OR_Hong Kong_ 2.48, 95% CI 1.80-3.44; OR_Taiwan_ 2.30, 95% CI 2.21-2.39) (see Table 3). The significance of other HBM constructs varied by country. Performing more handwashing practices was positively and significantly associated with perceived severity in the United States (OR_severity_ 1.33, 95% CI 1.09-1.61), perceived susceptibility in Mexico (OR_susceptibility_ 1.23, 95% CI 1.06-1.42) and Hong Kong (OR_susceptibility_ 1.44, 95% CI 1.11-1.87), and perceived susceptibility and perceived severity in Taiwan (OR_susceptibility_ 1.08, 95% CI 1.04-1.12; OR_severity_ 1.24, 95% CI 1.20-1.29). In the United States, Mexico, and Taiwan, those who perceived government measures for COVID-19 as restrictive were significantly less likely to handwash compared to those who perceived measures as appropriate or essential (OR_United States_ 0.41, 95% CI 0.30-0.55; OR_Mexico_ 0.65, 95% CI 0.50-0.86; OR_Taiwan_ 0.82, 95% CI 0.76-0.89). In the United States, non-White respondents were more likely to handwash compared to White or European respondents (OR_Hispanic/Latino or other_ 3.22, 95% CI 2.69-3.86; OR_Asian_ 2.76, 95% CI 1.89-4.04). Similar patterns of association for covariates persisted even when we used handwashing time (>20 seconds), another important COVID-19 handwashing behavior, as the binary outcome in the sensitivity analysis (see Multimedia Appendix 5).

Similar to handwashing, individuals with higher self-efficacy were also more likely to practice social distancing compared to those with lower self-efficacy (OR_United States_ 1.77, 95% CI 1.24-2.49; OR_Mexico_ 1.77, 95% CI 1.40-2.25; OR_Hong Kong_ 3.25, 95% CI 2.32-4.62; OR_Taiwan_ 2.58, 95% CI 2.47-2.68) (see Table 4). Social distancing was also positively associated with perceived severity in the United States (OR_severity_ 1.62, 95% CI 1.24-2.12), Mexico (OR_severity_ 1.29, 95% CI 1.11-1.50), and Taiwan (OR_severity_ 1.17, 95% CI 1.13-1.21). Similarly, in the United States, Mexico, and Taiwan, those who perceived government measures for COVID-19 as restrictive were significantly less likely to practice social distancing compared to those who perceived measures as appropriate or essential (OR_United States_ 0.52, 95% CI 0.36-0.76; OR_Mexico_ 0.65, 95% CI 0.49-0.85; OR_Taiwan_ 0.82, 95% CI 0.76-0.88). In the United States, family obligations and transportation needs were associated with fewer social distancing practices (OR_family_ 0.25, 95% CI 0.17-0.36; OR_transportation_ 0.25, 95% CI 0.11-0.57). In Hong Kong, those who had difficulty obtaining masks were more likely to socially distance (OR_masks_ 1.61, 95% CI 1.23-2.10), but those who had an essential job or transportation needs were less likely to socially distance (OR_essential job_ 0.66, 95% CI 0.48-0.89; OR_transportation_ 0.67, 95% CI 0.52-0.87). Similarly in Taiwan, those who had an essential job or transportation needs were less likely to socially distance (OR_essential job_ 0.71, 95% CI 0.68-0.74; OR_transportation_ 0.85, 95% CI 0.82-0.89).

**Table 3:** Multivariable model of health beliefs and handwashing practices by country.

Characteristic or construct and responses			United States, OR^a^ (95% CI)		*P* value		Mexico, OR (95% CI)		*P* value		Hong Kong, OR (95% CI)		*P* value		Taiwan, OR (95% CI)		*P* value
**Age group (years)**																	
	18-24	Reference				Reference				Reference				Reference			
	25-34	1.14 (0.65-2.03)		.70		1.15 (0.91-1.46)		.20		2.00 (1.14-3.55)		.02		1.44 (1.34-1.55)		<.001	
	35-44	1.24 (0.72-2.20)		.40		1.60 (1.25-2.04)		<.001		2.15 (1.24-3.80)		.007		2.24 (2.09-2.41)		<.001	
	45-59	1.91 (1.11-3.34)		.02		1.86 (1.47-2.35)		<.001		2.20 (1.29-3.82)		.004		2.66 (2.48-2.85)		<.001	
	60+	1.10 (0.63-1.95)		.70		1.76 (1.37-2.26)		<.001		1.16 (0.67-2.03)		.60		2.96 (2.76-3.18)		<.001	
**Gender**																	
	Female	Reference				Reference				Reference				Reference			
	Male	0.84 (0.71-1.00)		.05		1.00 (0.87-1.14)		.90		1.09 (0.85-1.40)		.50		0.69 (0.66-0.71)		<.001	
	Other^b^	1.52 (0.62-3.93)		.40		1.09 (0.56-2.11)		.80		1.73 (0.80-3.84)		.20		1.06 (0.96-1.17)		.02	
**Race or ethnicity**																	
	White or European	Reference				N/A^c^				N/A				N/A			
	Hispanic/Latino or other^d^	3.22 (2.69-3.86)		<.001		N/A		N/A		N/A		N/A		N/A		N/A	
	Asian	2.76 (1.89-4.04)		<.001		N/A		N/A		N/A		N/A		N/A		N/A	
**Education**																	
	Below college	Reference				Reference				Reference				Reference			
	College and above	0.99 (0.71-1.40)		.90		1.14 (0.95-1.36)		.20		1.13 (0.84-1.53)		.40		0.84 (0.80-0.88)		<.001	
**Perceived susceptibility of infection**																	
	Not likely/slightly likely	Reference				Reference				Reference				Reference			
	Moderately to very likely	1.12 (0.95-1.33)		.20		1.23 (1.06-1.42)		.006		1.44 (1.11-1.87)		.007		1.08 (1.04-1.12)		<.001	
**Perceived severity of COVID-19**																	
	Not afraid/slightly afraid	Reference				Reference				Reference				Reference			
	Moderately to very afraid	1.33 (1.09-1.61)		.005		1.06 (0.91-1.22)		.50		1.22 (0.90-1.65)		.20		1.24 (1.20-1.29)		<.001	
**Perceived benefits of handwashing measures**																	
	Unnecessarily restrictive/moderately restrictive	0.41 (0.30-0.55)		<.001		0.65 (0.50-0.86)		.002		0.65 (0.41-1.02)		.06		0.82 (0.76-0.89)		<.001	
	Appropriate/essential	Reference				Reference				Reference				Reference			
	Not enough	1.49 (1.23-1.82)		<.001		0.97 (0.84-1.12)		.70		0.77 (0.56-1.07)		.12		0.91 (0.86-0.96)		.001	
**Self-efficacy in carrying out handwashing measures**																	
	Not confident/moderately confident	Reference				Reference				Reference				Reference			
	Confident/very confident	1.58 (1.21-2.07)		<.001		1.54 (1.21-1.96)		<.001		2.48 (1.80-3.44)		<.001		2.30 (2.21-2.39)		<.001	
**Perceived barriers to following handwashing measures (reference is “no”)**																	
	Hand soap	0.73 (0.49-1.07)		.11		1.35 (0.98-1.87)		.07		7.59 (1.88-53.9)		.01		1.01 (0.81-1.27)		.90	
	Hand sanitizer	0.88 (0.74-1.03)		.11		1.01 (0.88-1.15)		.90		1.14 (0.74-1.77)		.50		0.86 (0.79-0.94)		<.001	
**Cues to action (reference is “no”)^e^**																	
	News source	0.77 (0.64-0.92)		.003		0.80 (0.70-0.92)		.001		0.97 (0.69-1.35)		.80		0.95 (0.91-0.99)		.02	
	Social media	0.60 (0.50-0.71)		<.001		0.77 (0.67-0.89)		<.001		0.79 (0.60-1.05)		.10		0.86 (0.83-0.89)		<.001	
	Central administration officials	1.32 (1.01-1.74)		.05		0.84 (0.73-0.98)		.02		1.27 (0.55-3.02)		.60		0.99 (0.95-1.03)		.70	
	Regional administration officials	0.63 (0.52-0.75)		<.001		0.83 (0.69-1.00)		.05		0.42 (0.22-0.77)		.007		1.08 (0.96-1.21)		.20	

^a^OR: odds ratio; models were run using weighted data, which were calculated by dividing the actual proportion of the country’s population by the proportion from the study’s sample, then renormalized for each country to ensure weighted and unweighted sample sizes were equal.

^b^Responses of *other* gender include individuals who chose nonbinary/third gender, prefer not to say, or other (<3% of total responses).

^c^N/A: not applicable; race or ethnicity was not adjusted for these countries as the majority identified as the same race or ethnicity.

^d^Responses of *other* race or ethnicity include individuals who are Black or African American, American Indian or Alaska Native, Native Hawaiian or other Pacific Islander, or other. Categories were collapsed due to low numbers (<2% of total responses).

^e^The top four media resources selected by respondents, when asked to pick their top three from the list, are shown.

**Table 4:** Multivariable model of health beliefs and social distancing practices by country.

Characteristic or construct and responses		United States, OR (95% CI)^a^	*P* value	Mexico, OR (95% CI)	*P* value	Hong Kong, OR (95% CI)	*P* value	Taiwan, OR (95% CI)	*P* value
**Age group (years)**									
	18-24	Reference		Reference		Reference		Reference	
	25-34	1.21 (0.52-2.66)	.60	0.70 (0.54-0.90)	.006	1.19 (0.68-2.08)	.50	1.39 (1.29-1.50)	<.001
	35-44	1.61 (0.71-3.53)	.20	0.69 (0.53-0.89)	.005	1.09 (0.63-1.89)	.80	1.82 (1.69-1.96)	<.001
	45-59	1.67 (0.72-3.65)	.20	0.67 (0.52-0.86)	.002	1.17 (0.69-1.99)	.60	1.93 (1.80-2.07)	<.001
	60+	3.30 (1.42-7.31)	.004	0.52 (0.40-0.68)	<.001	0.56 (0.32-0.97)	.04	1.89 (1.76-2.03)	<.001
**Gender**									
	Female	Reference		Reference		Reference		Reference	
	Male	1.36 (1.05-1.75)	.02	1.14 (0.99-1.31)	.07	1.04 (0.81-1.34)	.80	1.03 (1.00-1.07)	.07
	Other^b^	1.68 (0.47-8.23)	.50	1.02 (0.52-2.06)	.90	0.38 (0.15-0.87)	.03	0.92 (0.83-1.01)	.10
**Race or ethnicity**									
	White or European	Reference		N/A^c^		N/A		N/A	
	Hispanic/Latino or other^d^	0.46 (0.35-0.61)	<.001	N/A	N/A	N/A	N/A	N/A	N/A
	Asian	0.78 (0.44-1.42)	.40	N/A	N/A	N/A	N/A	N/A	N/A
**Education**									
	Below college	Reference		Reference		Reference		Reference	
	College and above	0.60 (0.36-0.96)	.04	1.32 (1.10-1.60)	.003	1.13 (0.83-1.54)	.40	1.17 (1.12-1.24)	<.001
**Reduced income**									
	No	Reference		Reference		Reference		Reference	
	Yes	0.60 (0.46-0.78)	<.001	0.95 (0.83-1.10)	.50	0.98 (0.74-1.30)	.90	1.07 (1.03-1.12)	.002
**Perceived susceptibility of infection**									
	Not likely/slightly likely	Reference		Reference		Reference		Reference	
	Moderately to very likely	1.11 (0.86-1.44)	.40	1.02 (0.88-1.19)	.80	0.76 (0.58-0.99)	.05	0.94 (0.90-0.97)	<.001
**Perceived severity of COVID-19**									
	Not afraid/slightly afraid	Reference		Reference		Reference		Reference	
	Moderately to very afraid	1.62 (1.24-2.12)	<.001	1.29 (1.11-1.50)	.001	1.34 (0.99-1.84)	.06	1.17 (1.13-1.21)	<.001
**Perceived benefits of social distancing measures**									
	Unnecessarily restrictive/moderately restrictive	0.52 (0.36-0.76)	<.001	0.65 (0.49-0.85)	.002	1.24 (0.79-1.96)	.40	0.82 (0.76-0.88)	<.001
	Appropriate/essential	Reference		Reference		Reference		Reference	
	Not enough	1.72 (1.24-2.42)	.001	1.22 (1.05-1.41)	.01	1.04 (0.74-1.46)	.80	1.05 (0.99-1.11)	.13
**Self-efficacy in carrying out social distancing measures**									
	Not confident/moderately confident	Reference		Reference		Reference		Reference	
	Confident/very confident	1.77 (1.24-2.49)	.001	1.77 (1.40-2.25)	<.001	3.25 (2.32-4.62)	<.001	2.58 (2.47-2.68)	<.001
**Perceived barriers to following social distancing measures (reference is “no”)**									
	Masks	0.95 (0.73-1.23)	.70	1.11 (0.96-1.28)	.20	1.61 (1.23-2.10)	<.001	0.92 (0.88-0.97)	.002
	Essential job	0.86 (0.61-1.23)	.40	0.85 (0.71-1.03)	.09	0.66 (0.48-0.89)	.007	0.71 (0.68-0.74)	<.001
	Family obligations	0.25 (0.17-0.36)	<.001	0.84 (0.70-1.01)	.06	0.80 (0.50-1.29)	.40	0.97 (0.92-1.04)	.40
	Transportation needs	0.25 (0.11-0.57)	<.001	0.78 (0.57-1.06)	.11	0.67 (0.52-0.87)	.002	0.85 (0.82-0.89)	<.001
**Cues to action (reference is “no”)^e^**									
	News source	1.58 (1.22-2.04)	<.001	0.96 (0.84-1.10)	.60	0.94 (0.66-1.33)	.70	0.93 (0.89-0.97)	<.001
	Social media	0.53 (0.41-0.68)	<.001	0.92 (0.80-1.07)	.30	0.85 (0.64-1.13)	.30	0.91 (0.88-0.95)	<.001
	Central administration officials	1.15 (0.78-1.75)	.50	1.04 (0.89-1.22)	.60	1.33 (0.56-3.13)	.50	1.15 (1.11-1.20)	<.001
	Regional administration officials	0.70 (0.53-0.92)	.01	1.20 (0.98-1.46)	.08	1.59 (0.90-2.83)	.11	1.07 (0.96-1.20)	.20

^a^OR: odds ratio; models were run using weighted data, which were calculated by dividing the actual proportion of the country’s population by the proportion from the study’s sample, then renormalized for each country to ensure weighted and unweighted sample sizes were equal.

^b^Responses of *other* gender include individuals who chose nonbinary/third gender, prefer not to say, or other (<3% of total responses).

^c^N/A: not applicable; race or ethnicity was not adjusted for these countries as the majority identified as the same race or ethnicity.

^d^Responses of *other* race or ethnicity include individuals who are Black or African American, American Indian or Alaska Native, Native Hawaiian or other Pacific Islander, or other. Categories were collapsed due to low numbers (<2% of total responses).

^e^The top four media resources selected by respondents, when asked to pick their top three from the list, are shown.

## Discussion

### Principal Findings

In this international study to examine COVID-19-related health behaviors using the HBM, we showed that respondents from the United States practiced the most social distancing, while those from Taiwan practiced the most handwashing. Despite these differences in health behaviors, self-efficacy was a significant predictor in all four countries. Our findings may be explained by the strictness and timing of government policies, the number of confirmed infection cases in each country, individual beliefs, and cultural context.

In the context of government interventions, Taiwan’s early border control, case identification, isolation of suspected cases, and resource allocation led to recommendations for social distancing, though not strictly enforced [27]. Similarly, Hong Kong’s early identification and strict quarantine of suspected cases resulted in regulations that prohibited large public gatherings but otherwise maintained regular activities [28,29]. On the other hand, 43 US states issued lockdown orders between mid-March and early April 2020, each lasting until the end of April at the minimum [30]. Mexico issued similar orders on March 26, 2020,with the strictest measures lasting until the end of May 2020 [31]. These varying degrees of strictness and timing of government interventions among the four countries may have attributed to increased social distancing in the United States and Mexico compared to Hong Kong and Taiwan.

Furthermore, at the start of our study period on April 11, 2020, the World Health Organization Situation Report recorded 1.6 million confirmed COVID-19 cases, with more than 99,000 deaths in over 200 countries and territories [32]. This included 461,275 confirmed cases in the United States, 3441 in Mexico, 1001 in Hong Kong, and 382 in Taiwan [26,32,33]. By the end of our study period on May 1, 2020, the numbers of cases and deaths had doubled worldwide [32]. The United States and Mexico saw a 124% and 417% increase in the number of confirmed COVID-19 cases, respectively, while Taiwan and Hong Kong reported only a 6.8% and 3.9% increase, respectively [26,32,33]. The rapid increase in confirmed cases in the United States and Mexico compared to Hong Kong and Taiwan likely also played a role in understanding handwashing and social distancing behaviors.

Among the HBM constructs, our study found that self-efficacy was the strongest positive predictor for both handwashing and social distancing in all countries. These findings were largely consistent with previous studies that examined preventative behaviors for cancers using the HBM [34,35]. Although a review of HBM studies suggested that the construct of perceived barriers was the best individual predictor across different types of studies and behaviors, self-efficacy can be seen as an important factor in overcoming the barriers to taking actions [17]. Perceived barriers were not significantly associated with hand hygiene, possibly because difficulties accessing water, soap, and hand sanitizer were less common among our survey respondents. For instance, Mexico is an upper-middle-income country and in some regions and communities these items might not be readily available; however, the population we reached through social media may be comparable to the populations we assessed from the other high-income countries [36]. For social distancing behaviors, having transportation needs was consistently associated with practicing less social distancing. This is especially relevant in Hong Kong and Taiwan, where public transportation is heavily utilized with their population densities of 6690 and 652 persons per square kilometer, respectively [37,38]. In their most densely populated districts, these numbers are even higher at 57,250 and 27,418 persons per square kilometer, respectively [37,39]. It is also important to note that we treated each perceived barrier as a unique covariate in the model to assess the most relevant barriers to social distancing, which may differ from other studies. Moreover, given that our respondents were mostly well-educated social media users, the true proportion of individuals with perceived barriers in our study was likely underestimated.

Previous studies have also suggested perceived susceptibility to be a good predictor for preventative behaviors [17]. In our study, perceived susceptibility was, overall, a significant positive predictor for practicing more handwashing. Perceived severity was also a strong predictor for both handwashing and social distancing in the United States and Taiwan, which may have been influenced by the worldwide news coverage and the strictness of government interventions. The associations found between perceived benefits and health behaviors may be tied to the timing of policies in each country and overall trust in the government. Cues to action, measured as types of media publicity, were not significant for predicting behavioral change in our model.

Modifiable factors that influence individual beliefs, such as culture and prior knowledge, are important to consider. In Hong Kong and Taiwan, wide adoption of preventative behaviors after the 2003 SARS outbreak may have better prepared residents for COVID-19, which may explain their greater sense of self-efficacy in handwashing and social distancing compared to other countries. Many residents were already taking regular individual actions, practicing good hand hygiene for infection control or wearing masks to counter air pollution when the pandemic hit. In fact, the study team received several emails from respondents in Taiwan and Hong Kong, noting that they had practiced handwashing prior to the pandemic because they were taught to do so as children. For this study, we were unable to statistically account for social factors and prior knowledge in our analyses, but future studies should consider including them into models to assess the influence of social and cultural factors on preventative health behaviors.

### Using Social Media for Recruitment During COVID-19

Our study may also provide insight into the effect of using social media recruitment strategies to reach a large audience. Given the rapidly evolving information, beliefs, and policies surrounding COVID-19, internet sampling allowed us to (1) capture real-time data simultaneously in different countries in a short time span, (2) reach a large number of participants in lockdown, and (3) overcome financial limitations [40]. The combination of boosting and sharing of social media posts allowed us to effectively target specific populations and locations while also reaching a larger audience, as was evident by the number of respondents from Taiwan. Our findings expand on recent COVID-19 studies from the United States and Taiwan that used similar methods to assess other COVID-19 attitudes, behaviors, and knowledge among different populations [41-43].

### Limitations

There are limitations to this study. Firstly, we used convenience sampling to recruit participants, which could have introduced potential sample selection bias. For example, we found an underrepresentation of populations with lower educational levels. This may have resulted in an overestimation of adherence rates and underestimation of perceived barriers. However, in multivariate analysis, education was not statistically associated with handwashing or social distancing practices. To best address the imbalances in our sample, we conducted poststratification weighting by age and gender, as well as race or ethnicity for the United States, to improve the generalizability of our results, although we understand that this does not make up for all of the differences [44]. Secondly, we had a disproportionately larger sample size in Taiwan relative to other countries [45]. However, since our main multivariable analyses were country specific, this would not likely affect the estimates found in other countries. Finally, there are weaknesses within the HBM itself. The HBM does not account for a person’s nonhealth-related beliefs or determinants that dictate a person’s acceptance of a health behavior. Health behaviors can also be learned through modeling as explained by other behavior change theories, such as the social cognitive theory (SCT); for instance, residents in Taiwan and Hong Kong might regularly wear masks and practice hand hygiene from observing those around them [46]. Self-efficacy, the strongest predictor in our study, is also known to play a large role in health behavior in the context of the SCT. However, we did not use SCT in our study because the theory’s heavy emphasis on the process of learning disregards an individual’s perception about COVID-19 as well as their motivations behind handwashing and social distancing behaviors. The collection of data on health beliefs, which the HBM encompasses, is important for the planning of interventions that can then be targeted to each country’s specific needs.

### Conclusions

Overall, our findings revealed that certain health belief constructs were independently associated with social distancing and handwashing behaviors. In the context of controlling the continued spread of COVID-19, self-efficacy is a significant predictor that can be easily targeted and modified by public health officials and educators. Policies and communications that address relevant health beliefs can facilitate adoption of necessary actions for preventing COVID-19.

## Appendix

Multimedia Appendix 1 Survey items.

Multimedia Appendix 2 Comparison of unweighted and weighted sample characteristics in the United States and Mexico relative to country population estimates.

Multimedia Appendix 3 Comparison of unweighted and weighted sample characteristics in Hong Kong and Taiwan relative to country population estimates.

Multimedia Appendix 4 Multivariable models assessing handwashing and social distancing practices by country.

Multimedia Appendix 5 Multivariable models of health beliefs and handwashing time (>20 seconds) by country.

## Abbreviations

HBM: health belief model
OR: odds ratio
SCT: social cognitive theory
