# Supplementary material for: Influence of Health Beliefs on Adherence to COVID-19 Preventative Practices: International, Social Media–Based Survey Study
Source: J Med Internet Res. 2021 Feb 26;23(2):e23720. doi: 10.2196/23720 (PMC7919844; doi:10.2196/23720)
Supplement: Multimedia Appendix 1 [file jmir_v23i2e23720_app1.docx]

| **Multimedia Appendix 1.** Survey items. | |
| --- | --- |
| Where do you currently live? |  |
| What do you think your risk is of getting infected with COVID-19? | Not likely  Slightly likely  Moderately likely  Likely  Very likely |
| How afraid are you of the COVID-19 pandemic? | Not afraid  Slightly afraid  Moderately afraid  Afraid  Very afraid |
| Do you currently wash your hands or use hand sanitizer in the following situations? | Immediately after entering your home  After grocery shopping  After touching non-household members  While in a public area  Before or after using your vehicle (e.g., car, motorcycle, bike, etc.)  After blowing your nose, or after coughing or sneezing in your hand  Before eating |
| Around how long do you currently wash your hands for? | Less than 10 seconds  Between 10-20 seconds  Over 20 seconds |
| Do you have difficulty getting any of the following items? (Check all that apply) | Running water  Soap  Hand sanitizer  Masks  None of the above |
| Are you doing any of the following? (Check all that apply) | Avoiding non-essential gatherings with non-household members  Keeping at least the recommended healthy distance from non-household members (e.g., 6 feet, 1.5 meters, 2 meters, or whatever your country recommends)  Avoiding close-contact with individuals (e.g., elderly, immunocompromised, underlying health conditions) at higher risk for severe illness from COVID-19  None of these |
| How has your income been affected by COVID-19? | I have lost my job  I am on unpaid leave  My income and/or hours have been reduced  I am on paid leave  No decrease in income  This question does not apply to me (e.g., retired, homemaker, not working) |
| Are you aware of measures to reduce the spread of COVID-19 (e.g., washing hands frequently, washing hands for 20 seconds, keeping the recommended healthy distance away from others, avoiding gatherings, covering coughs, etc.)? | I am aware of all/most  I am aware of some  I am not aware of any of these measures |
| How confident are you that you are able and willing to carry out these measures? | Not confident  Slightly confident  Moderately confident  Confident  Very confident |
| Have any barriers prevented you from adhering to these measures? (Check all that apply) | Having an essential job  Having financial pressure  Transportation  Family obligations or childcare  Housing insecurity or moving  Domestic violence  Needing medical care  Guidelines are unclear or confusing  None of the above  Other: |
| How do you feel about the government measures for COVID-19 in your area? | They are unnecessarily restrictive  They are moderately restrictive  They are appropriate  They are essential  They are not enough  I am not sure what the measures are in my area |
| What are your sources of information regarding COVID-19? (Choose up to 3) | Friends and family  Social media  Newspaper, news website, radio, or TV  Leading public health expert(s)  Medical institutions  Regional administration officials (e.g., state, county, provincial, city, town)  Central administration officials (e.g., federal or national)  A national public health organization (e.g., CDC)  World Health Organization (WHO)  Other: |
| How old are you? | 18-24 years old  25-34 years old  35-44 years old  45-59 years old  60-69 years old  70-79 years old  80+ years old |
| What gender do you identify with? | Male  Female  Non-binary/Third gender  Prefer not to say  Other: |
| What is your highest completed level of education? | Primary/Elementary school  Secondary/Middle school  High school  Vocational or technical school  College/University and above |
| [U.S.] What is your race/ethnicity? (Select all that apply) | White/Non-Hispanic or Latino  White/Hispanic or Latino  Black or African American  Asian  American Indian or Alaska Native  Native Hawaiian or other Pacific Islander  Other: |
| [All other] What is your race/ethnicity? (Select all that apply) | White or European  Black, African American, or African  Asian  Middle Eastern  Hispanic / Latino  Other: |
