# Supplementary material for: Influence of Health Beliefs on Adherence to COVID-19 Preventative Practices: International, Social Media–Based Survey Study
Source: J Med Internet Res. 2021 Feb 26;23(2):e23720. doi: 10.2196/23720 (PMC7919844; doi:10.2196/23720)
Supplement: Multimedia Appendix 2 [file jmir_v23i2e23720_app2.docx]

| **Appendix Table 2.** Comparison of unweighted and weighted sample characteristics in United States and Mexico relative to country population estimates^a^ | | | | | | | |
| --- | --- | --- | --- | --- | --- | --- | --- |
|  | **United States N = 3,070** | | | | **Mexico N = 3,946** | | |
| Characteristic and response | **Sample  Unweighted** | **Sample  Weighted** | **Population**^b^ | **Sample  Unweighted** | | **Sample  Weighted** | **Population**^b^ |
|  | n (%) | n (%) | % | n (%) | | n (%) | % |
| **Age group (years)** |  |  |  |  | |  |  |
| 18-24 | 71 (2.3) | 110 (3.6) | 8.7 | 199 (5.0) | | 507 (12.9) | 12.9 |
| 25-34 | 354 (11.6) | 519 (17.0) | 18.6 | 604 (15.3) | | 953 (24.2) | 24.3 |
| 35-44 | 593 (19.4) | 451 (14.7) | 16.9 | 1185 (30.1) | | 820 (20.8) | 20.8 |
| 45-59 | 1087 (35.5) | 963 (31.4) | 25.0 | 1341 (34.0) | | 977 (24.8) | 25.0 |
| 60+ | 955 (31.2) | 1019 (33.3) | 30.8 | 612 (15.5) | | 684 (17.3) | 17.4 |
| **Gender** |  |  |  |  | |  |  |
| Female | 2186 (71.5) | 1683 (55.0) | 51.0 | 2768 (70.3) | | 2031 (51.6) | 51.4 |
| Male | 837 (27.4) | 1351 (44.2) | 49.0 | 1130 (28.7) | | 1867 (47.4) | 48.6 |
| Other^c^ | 33 (1.1) | 25 (0.8) |  | 40 (1.0) | | 40 (1.0) |  |
| **Race or ethnicity** |  |  |  |  | |  |  |
| Asian | 2587 (84.7) | 158 (5.2) | 6.1 | N/A^d^ | | N/A | N/A |
| Hispanic/Latino or other^e^ | 239 (7.8) | 1520 (49.7) | 33.7 | N/A | | N/A | N/A |
| White/European | 227 (7.4) | 1379 (45.1) | 60.1 | N/A | | N/A | N/A |
| ^a^Weighted values were calculated by dividing the actual proportion of the country’s population by the proportion from the study’s sample, then renormalized for each country to ensure weighted and unweighted sample sizes were equal. Due to rounding and missing data (<5% for each item), the sum of n (%) for the sample weighted columns may not equal country’s total sample size.  ^b^Percentages were calculated out of total population who were 18 years and older  ^c^Responses of *other* gender include individuals who chose non-binary/third gender, prefer not to say, or other (<3% of total responses).  ^d^N/A: not applicable; race or ethnicity was not included in the weighting for these countries as the majority identified as the same race or ethnicity  ^e^Responses of *other* race/ethnicity include individuals who are Black or African American, American Indian or Alaska Native, Native Hawaiian or other Pacific Islander, or other. Categories were collapsed due to low numbers (<2% of total responses). | | | | | | | |
