# Supplementary material for: Influence of Health Beliefs on Adherence to COVID-19 Preventative Practices: International, Social Media–Based Survey Study
Source: J Med Internet Res. 2021 Feb 26;23(2):e23720. doi: 10.2196/23720 (PMC7919844; doi:10.2196/23720)
Supplement: Multimedia Appendix 4 [file jmir_v23i2e23720_app4.docx]

| **Appendix Table 4.** Multivariable models assessing handwashing and social distancing practices by country^a^ | | | | | |
| --- | --- | --- | --- | --- | --- |
|  | | Handwashing practices | | Social Distancing practices | |
|  | | OR^b^ | 95% CI^b^ | OR | 95% CI |
| **Age group (years)** | |  |  |  |  |
|  | 18-24 | 1.00 | (reference) | 1.00 | (reference) |
|  | 24-34 | **1.50** | **1.41, 1.61** | **1.33** | **1.24, 1.42** |
|  | 35-44 | **2.30** | **2.15, 2.45** | **1.80** | **1.68, 1.92** |
|  | 45-59 | **2.89** | **2.71, 3.08** | **2.07** | **1.94, 2.20** |
|  | 60+ | **3.27** | **3.07, 3.49** | **2.20** | **2.07, 2.34** |
| **Gender** | |  |  |  |  |
|  | Female | 1.00 | (reference) | 1.00 | (reference) |
|  | Male | **0.68** | **0.66, 0.71** | 0.98 | 0.95, 1.01 |
|  | Other^c^ | 1.08 | 0.98, 1.19 | 0.92 | 0.83, 1.00 |
| **Education** | |  |  |  |  |
|  | Below college | 1.00 | (reference) | 1.00 | (reference) |
|  | College and above | **0.90** | **0.86, 0.95** | **1.19** | **1.14, 1.25** |
| **Reduced income** | |  |  |  |  |
|  | No | 1.00 | (reference) | 1.00 | (reference) |
|  | Yes | **1.10** | **1.06, 1.15** | 0.96 | 0.92, 1.00 |
| **Country** | |  |  |  |  |
|  | Taiwan | 1.00 | (reference) | 1.00 | (reference) |
|  | Hong Kong | **0.67** | **0.59, 0.75** | **0.88** | **0.78, 0.99** |
|  | Mexico | **0.87** | **0.81, 0.93** | **2.17** | **2.02, 2.33** |
|  | United States | **0.50** | **0.46, 0.54** | **7.73** | **6.93, 8.66** |
| ^a^Models were run using weighted data, which were calculated by dividing the actual proportion of the country’s population by the proportion from the study’s sample, then renormalized for each country to ensure weighted and unweighted sample sizes were equal.  ^b^OR: odds ratio; CI: confidence interval. Bolded values denote statistical significance; *p* < 0.05  ^c^Responses of other gender include individuals who chose non-binary/third gender, prefer not to say, or other (<2% of total responses). | | | | | |
