# Supplementary material for: Influence of Health Beliefs on Adherence to COVID-19 Preventative Practices: International, Social Media–Based Survey Study
Source: J Med Internet Res. 2021 Feb 26;23(2):e23720. doi: 10.2196/23720 (PMC7919844; doi:10.2196/23720)
Supplement: Multimedia Appendix 5 [file jmir_v23i2e23720_app5.docx]

| **Appendix Table 5.** Multivariable models of health beliefs and handwashing time (>20 seconds) by country^a^ | | | | |
| --- | --- | --- | --- | --- |
| Characteristic or construct and responses | **United States** | **Mexico** | **Hong Kong** | **Taiwan** |
|  | **OR (95% CI)^b^** | **OR (95% CI)** | **OR (95% CI)** | **OR (95% CI)** |
| **Age group (years)** | Reference | Reference | Reference | Reference |
| 18-24 | 0.72 (0.41, 1.27) | **0.52 (0.41, 0.67)** | 0.89 (0.51, 1.58) | **0.86 (0.80, 0.94)** |
| 24-34 | **2.16 (1.26, 3.76)** | **0.57 (0.44, 0.74)** | 0.81 (0.46, 1.42) | **0.87 (0.81, 0.94)** |
| 35-44 | **1.80 (1.06, 3.12)** | **0.72 (0.56, 0.91)** | 0.74 (0.43, 1.28) | 0.93 (0.86, 1.00) |
| 45-59 | **2.13 (1.24, 3.70)** | **0.66 (0.51, 0.86)** | **0.51 (0.29, 0.91)** | **0.87 (0.80, 0.93)** |
| 60+ |  |  |  |  |
| **Gender** |  |  |  |  |
| Female | Reference | Reference | Reference | Reference |
| Male | **0.60 (0.51, 0.70)** | 0.90 (0.78, 1.04) | 1.03 (0.78, 1.35) | **0.91 (0.88, 0.94)** |
| Other^c^ | 0.82 (0.31, 2.03) | 0.60 (0.26, 1.25) | 1.16 (0.49, 2.63) | 0.90 (0.81, 1.00) |
| **Race or ethnicity** |  |  |  |  |
| White/European | Reference | N/A^c^ | N/A | N/A |
| Hispanic/Latino or other^d^ | **0.43 (0.36, 0.52)** | N/A | N/A | N/A |
| Asian | **0.53 (0.35, 0.78)** | N/A | N/A | N/A |
| **Education** |  |  |  |  |
| Below college | Reference | Reference | Reference | Reference |
| College and above | **2.03 (1.44, 2.89)** | 1.15 (0.95, 1.40) | **1.92 (1.36, 2.75)** | **1.1 (1.04, 1.16)** |
| **Perceived susceptibility of infection** |  |  |  |  |
| Not/Slightly likely | Reference | Reference | Reference | Reference |
| Moderately to very likely | 1.19 (1.01, 1.40) | **1.25 (1.07, 1.46)** | 1.16 (0.87, 1.54) | **1.06 (1.02, 1.11)** |
| **Perceived severity of COVID-19** |  |  |  |  |
| Not/Slightly afraid | Reference | Reference | Reference | Reference |
| Moderately to very afraid | **0.76 (0.63, 0.92)** | 0.88 (0.75, 1.03) | **1.55 (1.11, 2.19)** | **1.10 (1.06, 1.14)** |
| **Perceived benefits of handwashing measures** |  |  |  |  |
| Unnecessary/Moderately restrictive | **0.63 (0.47, 0.83)** | **0.67 (0.49, 0.91)** | 0.65 (0.39, 1.05) | **0.81 (0.74, 0.88)** |
| Appropriate/Essential | Reference | Reference | Reference | Reference |
| Not enough | **1.47 (1.21, 1.79)** | **1.11 (0.95, 1.29)** | 0.76 (0.53, 1.08) | **1.09 (1.02, 1.15)** |
| **Self-efficacy in carrying out handwashing measures** |  |  |  |  |
| Not/Moderately confident | Reference | Reference | Reference | Reference |
| Confident/Very confident | **2.29 (1.75, 3.01)** | **2.32 (1.73, 3.16)** | **3.22 (2.20, 4.83)** | **2.63 (2.51, 2.77)** |
| **Perceived barriers to following handwashing measures (ref = no)** |  |  |  |  |
| Hand soap | 0.91 (0.62, 1.31) | 0.72 (0.50, 1.03) | 0.32 (0.05, 1.29) | 0.89 (0.69, 1.14) |
| Hand sanitizer | 0.85 (0.72, 1.00) | **1.19 (1.04, 1.37)** | 1.08 (0.67, 1.71) | 0.95 (0.86, 1.04) |
| **Cues to action (ref = no)^e^** |  |  |  |  |
| News source | 1.03 (0.86, 1.23) | **0.70 (0.61, 0.81)** | 1.26 (0.87, 1.85) | **0.77 (0.73, 0.80)** |
| Social media | **0.66 (0.55, 0.78)** | **0.53  (0.45, 0.61)** | **1.42 (1.05, 1.95)** | **0.78 (0.75, 0.81)** |
| Central administration officials | 0.87 (0.66, 1.15) | 1.07 (0.91, 1.24) | **5.37 (2.25, 13.4)** | **1.07 (1.03, 1.12)** |
| Regional administration officials | **0.55 (0.46, 0.65)** | 1.11 (0.91, 1.35) | **2.24 (1.26, 3.98)** | 1.01 (0.90, 1.14) |
| ^a^OR: odds ratio; models were run using weighted data, which were calculated by dividing the actual proportion of the country’s population by the proportion from the study’s sample, then renormalized for each country to ensure weighted and unweighted sample sizes were equal. ^b^Responses of *other* gender include individuals who chose non-binary/third gender, prefer not to say, or other (<3% of total responses). ^c^N/A: not applicable; race or ethnicity was not adjusted for these countries as the majority identified as the same race or ethnicity ^d^Responses of *other* race/ethnicity include individuals who are Black or African American, American Indian or Alaska Native, Native Hawaiian or other Pacific Islander, or other. Categories were collapsed due to low numbers (<2% of total responses). ^e^The top four media resources selected by respondents, when asked to pick their top three from the list, are shown. | | | | |
